# Solitary fibrous tumor of the parotid gland: Case report

**DOI:** 10.4317/jced.51103

**Published:** 2013-10-01

**Authors:** Alexandre A. Sousa, Giovanna R. Souto, Isabela A. Sousa, Ricardo A. Mesquita, Ricardo S. Gomez, Bruno C. Jham

**Affiliations:** 1 MD, PhD. School of Medicine, Federal University of Minas Gerais, Av. Alfredo Balena, 190, Sta Efigênia, Belo Horizonte, Minas Gerais, Brazil; 2 DDS, MS. School of Dentistry, Federal University of Minas Gerais, Av. Antonio Carlos, 6627, Pampulha, Belo Horizonte, Minas Gerais, Brazil; 3 DDS, School of Medicine, Federal University of Minas Gerais, Av. Alfredo Balena, 190, Sta Efigênia, Belo Horizonte, Minas Gerais, Brazil; 4 DDS, PhD. School of Dentistry, Federal University of Minas Gerais, Av. Antonio Carlos, 6627, Pampulha, Belo Horizonte, Minas Gerais, Brazil; 5 DDS, PhD. College of Dental Medicine – Illinois, Midwestern University, 555 31st Street, Science Hall 211-R, Downers Grove, Illinois, USA

## Abstract

Solitary fibrous tumor (SFT) is a rare spindle cell neoplasm that usually develops in the pleura and peritoneum. The head and neck region is involved in only 6% of the cases. Involvement of the parotid gland is a rare phenomenon, with only 24 cases reported in the literature. The aim of this study is to report an additional case of SFT affecting the parotid gland, and to review the literature on previously reported cases. The patient was a 42-year-old male with a 4-cm, fibro-elastic, movable, painless nodule in the inferior lobe of the parotid gland. The lesion was surgically excised and, following histopathological and immunohistochemical analysis, a diagnosis of SFT was rendered. The patient has been followed-up for ten months, with no signs of recurrence. Clinical, histopathological, immunohistochemical and treatment aspects of the tumor are discussed.

** Key words:**Solitary fibrous tumor, parotid gland, case report.

## Introduction

Solitary fibrous tumor (SFT) is a rare spindle cell neoplasm that usually develops in the pleura and peritoneum (1). Although the lesion has also been described in other tissues not associated with serosal surfaces, the head and neck is involved in only 6% of the cases (2). Involvement of the parotid gland is even a rarer phenomenon, with only 24 cases reported in the literature (3-5). The aim of this study is to report an additional case of SFT affecting the parotid gland, and to review the literature on previously reported cases. Clinical, histopathological, immunohistochemical and treatment aspects of the tumor are discussed.

## Case Report

**Figure 1:**
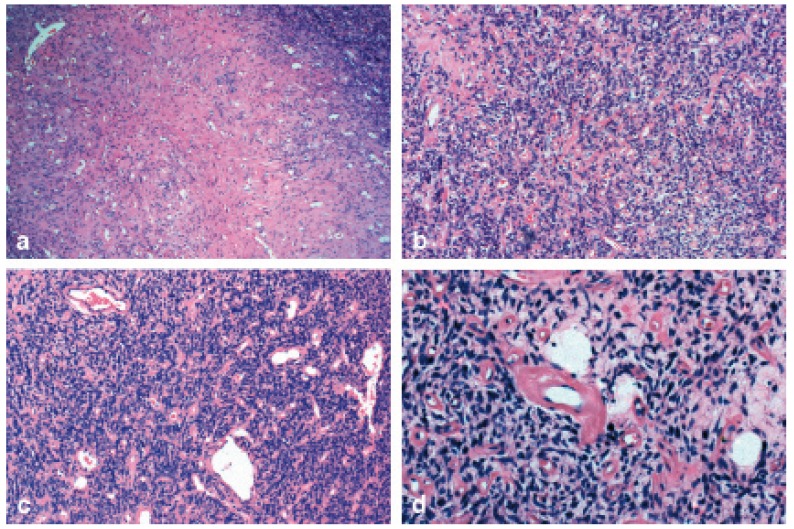
Histopathological findings. a) A heterogenous proliferation without a defined architectural pattern and alternating areas of hyper- and hypocellularity was observed; b) Intercellular collagen deposition was noted within the spindle cell proliferation; c) Hemangioperycytoma-like area of the tumor; d) Hyalinization of vessels was also noted.

**Figure 2:**
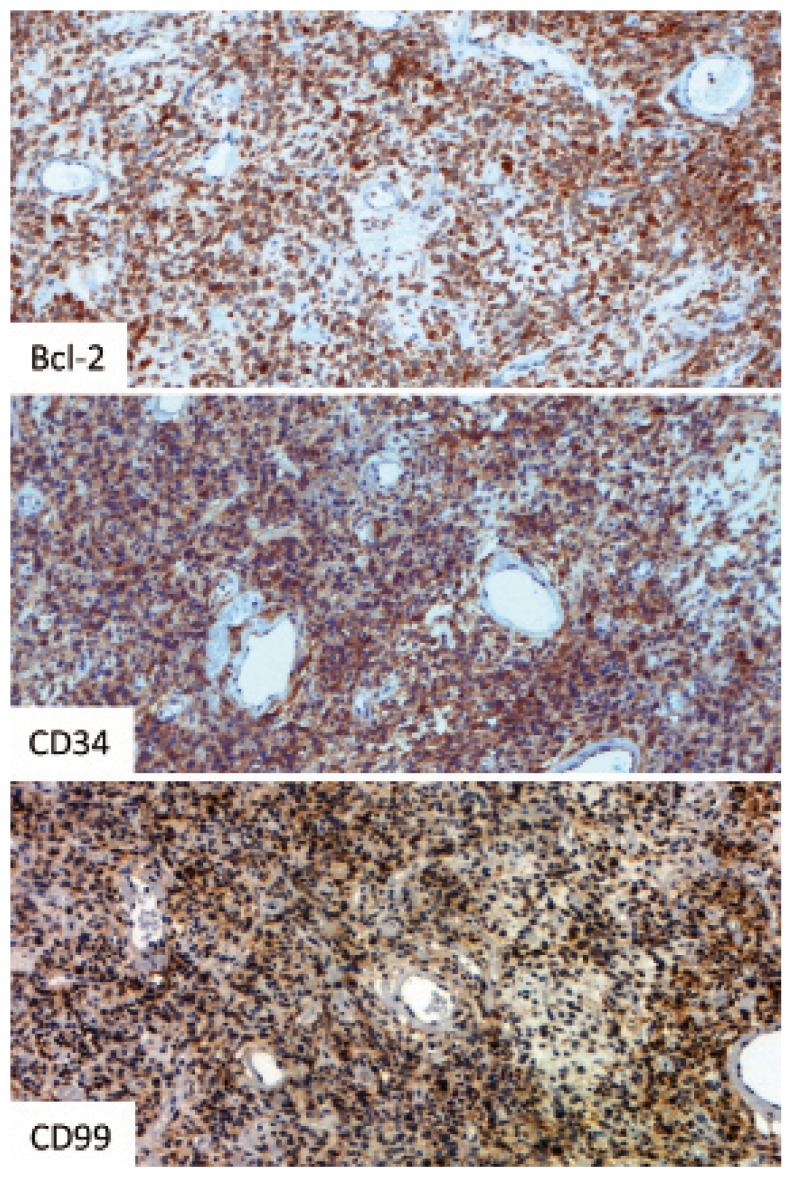
Immunohistochemical positivity was noted for bcl-2, CD34 and CD99.

A 42-year-old male was seen with a nodule on his left parotid, with evolution of two months. Medical history was non-contributory. Extra-oral clinical examination showed a 4-cm, fibro-elastic, movable, painless nodule in the inferior lobe of the parotid gland. With the clinical impression of a benign salivary gland tumor, the patient was submitted to left partial parotidectomy, with conservation of the facial nerve, and dismissed 12 hours after surgery. Gross examination showed a 7.5 x 4.5 x 3.0 cm parotid gland, weighing 25g with a soft, well-defined, 4.0 x 3.0 x 3.0 cm mass. The lesion was light-brown and hemorrhagic. Microscopic examination showed a well-circumscribed, lobulated proliferation of spindle cells. The lesion was heterogenous without a defined architectural pattern, with alternating areas of hyper- and hypocellularity (Fig. 1). Intercellular collagen deposition (Fig. 1) and multifocal cystic degeneration were observed. The lesion was highly vascular, with hemangioperycytoma-like areas (Fig. 1). Hyalinization of vessels was also noted (Fig. 1). Atypical mitoses, necrosis, pleomorphism or other signs of malignancy were absent. Immunohistochemical analysis showed the lesional cells were positive for CD34, CD99 and bcl-2 (Fig. 2), and negative for CAM-5.2, SMA and S100. Based on histopathological and immunohistochemical results, the final diagnosis was solitary fibrous tumor. The patient has been followed-up for ten months, with no signs of recurrence.

## Discussion

SFT is exceptionally rare in the parotid gland. In 2011, Bauer et al. (3) reviewed the literature on 21 cases and reported an additional lesion. In 2012, two additional cases were published (4,5). Thus, including the case here in described, only 25 cases have been reported to date. Initially, it was incorrectly believed that the lesion was of mesothelial origin (6). Subsequently, immunohistochemical and ultrastructural studies showed that SFT is most likely derived from adult mesenchymal stem cells (7). In the parotid gland, the tumor presumably originates from mesenchymal cells of the parenchyma (8).

Our patient was a 42-year-old male and reported first noticing the lesion two months before. This is in accordance with the literature, which indicates a slight male predominance (1.2:1 F:M ratio) and a mean age of 49.8 years. However, it is important to note that SFT affects patients of all ages, ranging from 11 to 79 years. SFT is more common on the left parotid gland, as seen in our case. Of the 22 reported cases which specified the side of the lesion, 14 were in the left side, while 8 were in the right side (1.75:1 ratio). The lesion grows slowly and may achieve significant sizes. Based on review of 25 cases, duration of symptoms ranged from 4 to 120 months. The mean size on diagnosis was 4.4 cm, with some reported cases reaching 12 cm (range 1–12 cm). Clinically, parotid SFT may mimic a variety of diseases and the differential diagnosis includes conditions that cause unilateral parotid enlargement, such as salivary gland tumors, mesenchymal neoplasms, lymphomas, Sjogren’s syndrome, sarcoidosis, sialadenosis and infections (3-5).  Table 1 summarizes the demographic and clinical information of all previously published cases of parotid SFT, including the case here in described.

**Table 1:**
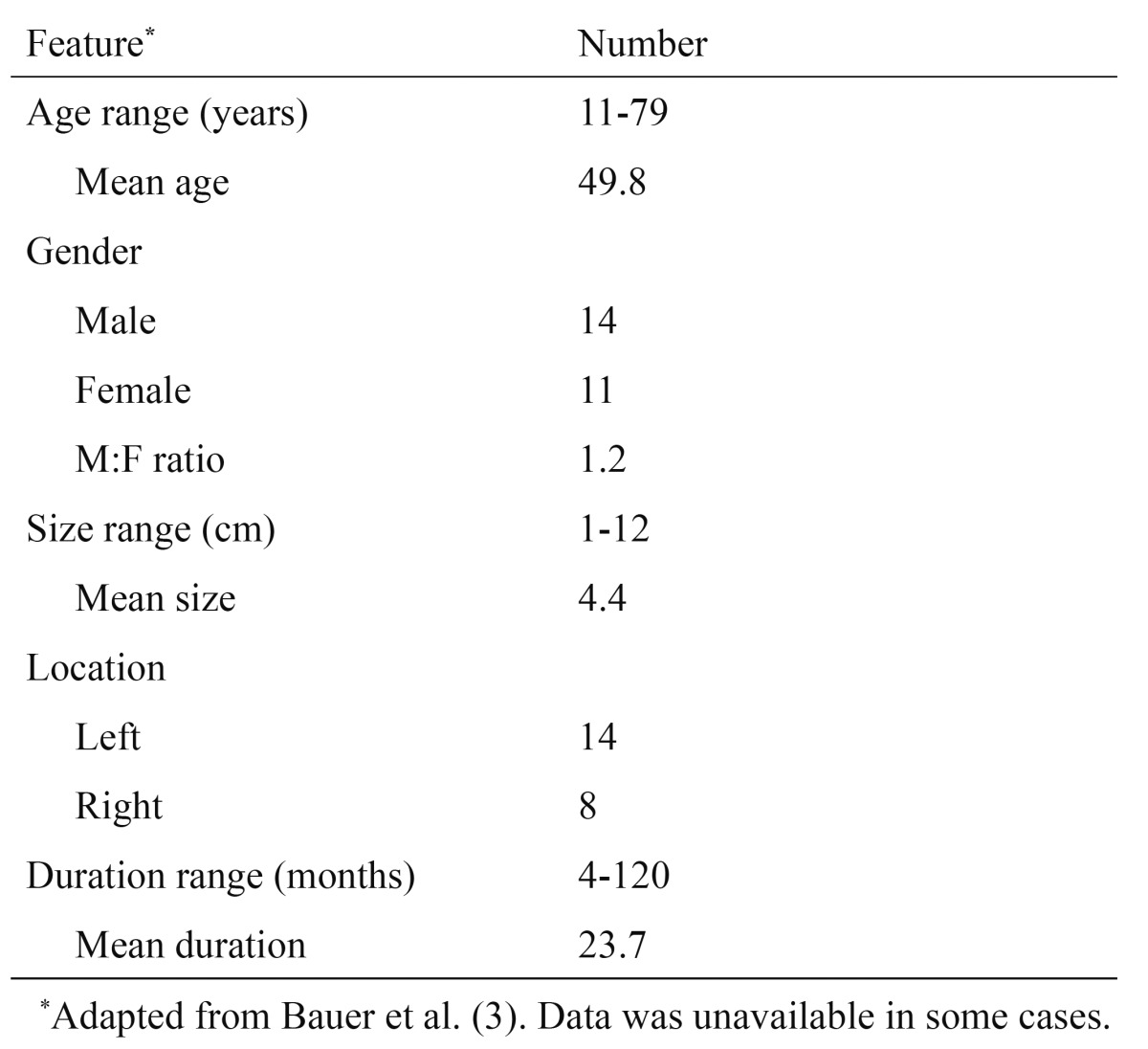
Demographic, clinical and immunohistochemical profile of 25 reported SFT cases.

Grossly, parotid SFT present as firm, white-tan or gray, partially or fully encapsulated, well-circumscribed lesions (3). Microscopically, round to spindled cells, arranged in fascicular, storiform or fibrosarcoma-like patterns, are observed. Cells have round to oval, centrally placed nuclei, with open, vesicular nuclear chromatin distribution. The stroma shows numerous medium-sized ramifying vessels, which may have thickened or hyalinized walls. There are a variety of histologic features accepted as diagnostic criteria, including alternating hypercellular and hypocellular to fibrous areas. In addition, a number of additional features can be seen, including stromal myxoid change, inflammatory cells, especially mast cells, and isolated multinucleated stromal tumor giant cells (3). Due to SFT’s wide spectrum of histologic features, there is a broad differential diagnosis. There is a significant morphologic overlap between SFT and hemangiopericytoma (HPC), and experts have questioned the concept of the latter. Indeed, Gengler and Guillou (9) suggested that most tumors that had been diagnosed HPC in the past were not truly of pericytic origin, but instead a cellular variant of SFT. Lesions to be considered in the differential diagnosis include cellular pleomorphic adenoma, myoepithelioma, schwannoma, neurofibroma, benign fibrous histiocytoma, nodular fasciitis, fibromatosis, myofibroblastoma, meningioma, fibrosarcoma, spindle cell squamous cell carcinoma, spindle cell melanoma, Kaposi sarcoma and monophasic synovial sarcoma (3). Thus, immunohistochemistry is often required to reach the final diagnosis. CD34 is the most important and sensitive marker for the diagnosis of SFT, although it may not be positive in all cases. Virtually all cases of SFT stain with vimentin and the vast majority is positive for CD34, bcl-2 and CD99 (3). In previous reports, 13/13, 19/21, 8/9 and 5/6 parotid SFT were positive for vimentin, CD34, bcl-2 and CD99, respectively. Parotid SFT stained negatively for S100, SMA and CAM 5.2 in 16/16, 14/14 and 7/7 previously described cases, respectively (3-5).

SFT are generally treated by complete local surgical excision. Because SFTs are often highly vascular, profuse bleeding is possible when resecting the tumor (10). Some authors have reported favorable results with postoperative chemotherapy and radiotherapy after surgery in cases whith incomplete resection (11). Despite a short follow-up period in previously reported cases, recurrence was not a common finding (3). Nonetheless, long-term follow-up with clinical and imaging examinations (ultrasonography and/or computed tomography) for at least 3 to 5 years has been recommended (5).

Of the 25 cases previously reported, three were diagnosed as malignant SFT. Grossly, two tumors were well-circumscribed (3). Tumors possessing histologically malignant features such as high cellularity, pleomorphism, necrosis, high mitotic rate, and/or infiltrative margins are more likely to behave aggressively. In general, more than six mitoses per 10 high-power fields suggest a malignant tumor. However, it should be noted that histologic features do not reliably predict aggressive clinical behavior (9,12,13). Immunohistochemically, malignant SFT tends to show reduced CD34 reactivity when compared to benign tumors (9). Regarding treatment, there is currently no evidence that malignant SFTs require additional treatment beyond excision, as long as the lesion is completely excised (14,15).
